# A novel technique for the treatment of post operative retro-rectal haematoma: two case reports

**DOI:** 10.1186/1757-1626-3-42

**Published:** 2010-02-01

**Authors:** Maruthesh Gowda Chikkappa, Charles Morrison, Jay Gokhale, Ralph Antrum

**Affiliations:** 1 Department of General Surgery, Bradford Royal Infirmary, Bradford, BD9 6RJ, UK

## Abstract

Rectal bleeding following any form of rectal surgery is a well recognised complication 1, 2, 3 & 4. However retro-rectal bleeding and tracking which then presents as rectal bleeding has not been reported in the literature. We describe a novel way of dealing with this technically difficult post-operative complication.

We present two cases of significant rectal bleeding (one following STARR procedure and other after Delormes procedure). Both patients had to be taken back to theatre due to continuing, significant bleeding. Examination under anaesthesia on both occasions revealed a posterior boggy swelling, with an opening which admitted a finger. In both cases clots were evacuated and a corrugated drain was inserted in to the retro rectal space.

The authors believe that following any form of rectal surgery, retro-rectal bleeding with tracking can present as rectal bleeding. Treatment in the form of EUA and insertion of corrugated helped to resolve the problem.

We present both cases and literature review of the complications of stapled haemorrhoidopexy.

## Introduction

Post operative rectal bleeding after stapled transanal rectal resection (STARR) has been widely reported. Management of this common but potentially life threatening postoperative complication remains a technically difficult challenge. We describe a novel way of treating this complication.

## Case presentation

### Case report 1

A 49-years-old Caucasian, British lady underwent stapled transanal rectal resection (STARR) procedure for second degree haemorrhoids and mucosal prolapse confirmed at the time of operation. Patient was discharged same day with analgesics and laxatives.

Patient presented following day complaining of inability to urinate and was found to have residual of 1300 ml for which she was catheterised and hospitalised. On examinations patient was haemodynamically stable but noted to have haemoglobin (Hb) of 9.3 gms/lt down from 14.3 gms/lt recorded pre operatively. Ultrasound (USS) of abdomen and pelvis requested after review by urologist was normal. On the following day blood tests revealed Hb of 8.1 and digital rectal examination (DRE) done by the colorectal consultant revealed extensive bruising around perineum, both sides of labia and thickening of rectal wall with old blood on gloves. Staple line was not felt, as examination could not be completed due to discomfort. Oral cefadrine and metronidazole were prescribed. Vital signs were blood pressure of 96/57, pulse of 97, respiratory rate of 24 and saturation of 97% and temperature of 36.5°.

Upon review by the operating surgeon following day the patient was taken for examination under anaesthetic (EUA). At the time of EUA, patient was found to have boggy mass posteriorly with small defect at 6 o' clock position below staple line through which blood clots were coming out. Staple line was intact. Blood was evacuated through the defect. Post operative plan was to continue antibiotics and repeat EUA 3 days later. In the interim Hb remained stable.

Repeat EUA confirmed the defect which had been previously noted. Again clots were evacuated and the cavity was irrigated with 700 mls of saline. This time a corrugated drain was inserted. Antibiotics were changed to Piptazobactam because of concerns of infection, on the advice of microbiologist and 2 units of packed cells were transfused because of sings of continued bleeding, pulse rate of 112 and blood pressure of 87/56 intra-operatively.

Patient was discharged home after full recovery a week later and remained well on follow up.

### Case report 2

An 84-years-old Caucasian, British lady had a routine Delormes procedure and stayed overnight because of social reasons. An episode of per rectal (PR) bleeding with low blood pressure of 88/56, pulse of 72, respiratory rate of 18, saturation of 99% and temperature of 37° was reported next day. Hb remained stable at 9.3 gms/lt and was kept in hospital for observation. Two days later another episode of PR bleeding associated with shortness of breath was reported. Vital measurements at this time were blood pressure of 98/66, pulse of 90, respiratory rate of 26, saturation of 94% and temperature was 36°

Digital rectal examination (DRE) at that time showed extensive bruising and old blood coming from rectum. Electrocardiogram and chest radiograph did not show any abnormality and again Hb remained stable. After another episode of PR bleeding on next day patient was taken for EUA. At the time of EUA, she was found to have boggy swelling at 9 o' clock position with small defect. Suture line was intact distal to it.

From the experience of the first case, cavity was irrigated with normal saline and corrugated drain was inserted. Oral antibiotics were started. Drain was taken out 4 days later and patient was allowed home the following day.

## Discussion

Stapled haemorrhoidopexy is a widely advocated operation because of shorter hospital stay and less discomfort but it is associated with different complications compared to traditional haemorrhoidectomy [1].

Post operative rectal bleeding after any rectal procedure is common and incidence varies from 0.01% to 25% [1]. and is technically difficult problem to deal with. Staple line bleeding points needing haemostasis at the time of surgery [1], have been reported since the original description of stapled haemorrhoidopexy by Longo at 6^th ^World Congress of Endoscopic Surgery in 1998. The need to take patient back to theatre for bleeding within a few hours of stapled haemorrhoidopexy is reported to be around 3% [2]. In these cases bleeding is usually from arteriolar vessels of the submucosa, which could be stopped by local haemostasis [3]. It could also be due to staples not achieving proper haemostasis or folded mucosa in the staple line.

Post operative bleeding in these cases is potentially a very serious complication, which may be under-estimated. Timely and appropriate assessment and possible intervention by an experienced surgeon is needed. Serious complications reported include continued rectal bleeding up to 12 months [4], perirectal haematoma leading to hypovolumic shock [5], uncontrollable intra abdominal bleeding needing low anterior resection [6], faecal peritonitis from rectal perforation [7] and death [8].

Though flavonoids were initially advocated for the reduction of bleeding and pain after stapled haemorrhoidectomy, Mlakar B [9], showed they did not make significant difference. Complications due to staple line failure leading either to hemorrhage or leakage can nowadays be treated with coagulation materials such as glues, however large trials rest to prove their value [10].

Author thinks that retro rectal bleeding tracks down in retro rectal space and form boggy mass which opens up and presents as rectal bleeding. This kind of bleeding is better treated by the novel technique described.

## Conclusion

Meticulous haemostasis achieved at the time of surgery is the most important factor in preventing post operative bleeding. In our report we have described a novel technique for the management of a not infrequent post operative complication that can be technically difficult to treat and have potentially serious consequences.

## Consent

Written informed consent was obtained from the patient for publication of this case report and accompanying images. A copy of the written consent is available for review by the Editor-in-Chief of this journal.

## Competing interests

The authors declare that they have no competing interests.

## Authors' contributions

GCM and CM wrote the case report including references. JAG and RA read suggested the changes. All authors read and agreed the final manuscript.
